# Expression of human Kallikrein 14 (KLK14) in breast cancer is associated with higher tumour grades and positive nodal status

**DOI:** 10.1038/sj.bjc.6602956

**Published:** 2006-01-24

**Authors:** F Fritzsche, T Gansukh, C A Borgoño, M Burkhardt, S Pahl, E Mayordomo, K-J Winzer, W Weichert, C Denkert, K Jung, C Stephan, M Dietel, E P Diamandis, E Dahl, G Kristiansen

**Affiliations:** 1 Institute of Pathology, Department of Urology, Charité Universitätsmedizin, Berlin, Germany; 2 Department of Pathology and Laboratory Medicine, Mount Sinai Hospital, Toronto, Canada; 3 Department of Laboratory Medicine and Pathobiology, University of Toronto, Toronto, Canada; 4 Breast Cancer Center, Charité Universitätsmedizin, Berlin, Germany; 5 Department of Urology, Charité Universitätsmedizin, Berlin, Germany; 6 Institute of Pathology, University Hospital of the RWTH Aachen, Aachen, Germany

**Keywords:** breast cancer, kallikrein, prognostic marker, immunohistochemistry, human kallikrein 14, serine protease

## Abstract

Human kallikrein 14 (KLK14) is a steroid hormone-regulated member of the tissue kallikrein family of serine proteases, for which a prognostic and diagnostic value in breast cancer has been suggested. To further characterise the value of KLK14 as a breast tumour marker, we have carefully analysed KLK14 expression in normal breast tissue and breast cancer both on the RNA level by real-time RT-PCR (*n*=39), and on the protein level (*n*=127) using a KLK14-specific antibody for immunohistochemistry. We correlated KLK14 protein expression data with available clinico-pathological parameters (mean follow-up time was 55 months) including patient prognosis. KLK14 RNA expression as quantified by real-time RT-PCR was significantly more abundant in breast tumours compared to normal breast tissue (*P*=0.027), an issue that had not been clarified recently. Concordantly with the RNA data, cytoplasmic KLK14 protein expression was significantly higher in invasive breast carcinomas compared to normal breast tissues (*P*=0.003). Furthermore, KLK14 protein expression was associated with higher tumour grade (*P*=0.041) and positive nodal status (*P*=0.045) but was not significantly associated with shortened disease-free or overall patient survival time in univariate analyses. We conclude that KLK14 is clearly overexpressed in breast cancer in comparison to normal breast tissues and is positively associated with conventional parameters of tumour aggressiveness, but due to a missing association with survival times, the use of KLK14 immunohistochemistry as a prognostic marker in breast cancer is questionable.

Human tissue kallikreins comprise a family of 15 secreted serine proteases, encoded by a multigene cluster (*KLK* genes) on chromosome 19q13.4 (Diamandis *et al*, 2000; Borgono *et al*, 2004; Clements *et al*, 2004). This family includes the most important serum tumour marker in clinical medicine for the early detection, risk stratification, clinical staging, and monitoring of prostate cancer, kallikrein 3 (KLK3), which is better known as prostate-specific antigen (PSA) (Diamandis, 1998). In recent years, many kallikreins in addition to KLK3/PSA, such as KLK2, KLK4, KLK5, KLK6, KLK7, KLK8, KLK9, KLK10, KLK11, KLK13, and KLK15 were found to be candidate biomarkers for several endocrine-related malignancies, including breast, ovarian and prostate cancers, demonstrating the immense clinical applicability of this family (Diamandis and Yousef, 2002; Borgono and Diamandis, 2004). With respect to breast cancer in particular, several kallikrein family members, namely KLK3 (Yu *et al*, 1996), KLK5 (Yousef *et al*, 2003c), KLK6 (Anisowicz *et al*, 1996), KLK10 (Liu *et al*, 1996; Dhar *et al*, 2001), KLK12 (Yousef *et al*, 2000b), KLK13 (Yousef *et al*, 2000a), and KLK14 (Borgono *et al*, 2003) were shown to be differentially expressed at the mRNA or protein levels within the cancerous tissues or serum of such patients. Furthermore, a number of preliminary analyses suggest that certain kallikreins including KLK3 (Yu *et al*, 1995; Yu *et al*, 1998; Foekens *et al*, 1999), KLK5 (Yousef *et al*, 2002b, 2003c; Diamandis *et al*, 2003), KLK7 (Talieri *et al*, 2004), KLK9 (Yousef *et al*, 2003a), KLK10 (Luo *et al*, 2002), KLK13 (Chang *et al*, 2002), KLK14 (Borgono *et al*, 2003), KLK15 (Yousef *et al*, 2002c) may possess clinical utility as diagnostic, prognostic or predictive breast cancer biomarkers. For recent reviews on the human kallikrein family please see references (Diamandis and Yousef, 2002; Borgono and Diamandis, 2004; Borgono *et al*, 2004; Clements *et al*, 2004).

Human kallikrein gene 14 (formerly known as *KLK-L6*) was cloned in 2001 via the positional candidate approach (Hooper *et al*, 2001; Yousef *et al*, 2001). The *KLK14* gene is under steroid hormone regulation and is predominantly expressed in endocrine tissues (breast, prostate, ovary, testes) as detected on the mRNA and protein level (Yousef *et al*, 2001; Borgono *et al*, 2003; Yousef *et al*, 2003b, 2003d). A recent study by Borgono *et al* (2003) revealed that KLK14 protein levels are elevated in the serum of a proportion of patients with breast cancer, implicating KLK14 as a potential marker for breast cancer. However, conflicting data have been obtained regarding the question whether KLK14 is overexpressed in breast tumours compared to normal breast tissue. Although one study reported loss of KLK14 expression in 21 of 25 analysed breast tumours in comparison to normal breast tissue (Yousef *et al*, 2001), another study demonstrated abundant KLK14 expression in tumours and an association of high KLK14 expression with advanced disease (Yousef *et al*, 2002a). Thus, the presently available data leaves the question open whether KLK14 is a suitable biomarker for human breast cancer. Therefore, the main focus of the present study was to carefully and quantitatively analyse KLK14 protein expression in a large collection of human breast normal and tumour specimens to further evaluate the potential value of KLK14 for breast cancer prognosis.

## MATERIALS AND METHODS

### Patients

For immunohistochemistry, our study included 127 patients diagnosed with primary breast cancer at the Institute of Pathology, Charité Universitätsmedizin, Berlin, between 1991 and 1997. Patient age at the time of diagnosis ranged from 30 to 87 with a median of 57 years (mean 59). Follow-up data including overall survival and disease recurrence or progression times were available for all cases. The average observation time for overall survival was 55 months for patients still alive at the time of analysis, and ranged from 1 to 130 months. Twenty-two patients (17%) died during follow-up and 43 patients (34%) experienced disease progression defined by either metastatic or local recurrent disease.

Adjuvant therapy was administered as follows: The first group received either no or local therapy/radiotherapy (29 cases), or systemic therapy excluding tamoxifen (20 cases). The second group had received tamoxifen with or without an additional systemic or local therapy (70 cases). For eight patients, no data on adjuvant therapy was available.

The selection of cases for this study was based on availability of tissue. Cases were not stratified for any known preoperative or pathological prognostic factors. Cases with systemic disease (M1) at the time of diagnosis were excluded. Tumour histology was determined according to the criteria of the World Health Organization (Tavassoli and Devilee, 2003), whereas disease stage was assessed according to UICC (Wittekind *et al*, 2002). Tumours were graded according to Bloom and Richardson, as modified by Elston and Ellis (1993). Data regarding oestrogen receptor status and the expression of c-erbB2 were taken from archival pathology reports. Oestrogen receptor positivity was defined as an immunoreactive score (IRS) >3. Overexpression of c-erbB2 was defined according to the clinical trial assay (2+, 3+) as recommended in the Hercep-test (DAKO). The clinicopathological characteristics of the patients/tumours are described in Table 1.

### RNA extraction from formalin-fixed paraffin-embedded breast normal and tumour tissue

Archival formalin-fixed paraffin-embedded tissue from 25 breast cancer specimens and 14 normal breast tissue specimens were obtained from patients diagnosed at the Institute of Pathology of the University Hospital of Aachen, Germany. For each formalin-fixed paraffin-embedded tissue specimen six 4-*μ*m thick tissue sections were cut with a microtome (Leica, Germany) and transferred to a water bath filled with DEPC-treated water. Sections were mounted on standard glass slides and dried for 1 h at 60°C. Sections were deparaffinised and rehydrated as follows: 2 × 15 min in xylene, 2 × 15 min in 100% ethanol, and short rinses in 96, 70 and 50% ethanol followed by emersion in distilled water. All tumour sections were manually microdissected to ensure a minimum of 80% tumour cells. Tissue material was transferred to a microcentrifuge tube and RNA was extracted according to the Trizol protocol supplied by the manufacturer (Life Technologies, Mannheim, Germany).

### Quantitative RT-PCR

*KLK14* mRNA-expression was analysed with the LightCycler® system (Roche Diagnostics, Germany) in archival formalin-fixed paraffin-embedded breast cancer and normal breast tissue specimens. *β*-Actin *mRNA* was used as reference. Primers used in this study are presented in Table 2. Real-time RT-PCR was carried out with Fast Start DNA master hybridisation probes (Roche Molecular Biochemicals, Germany). The conditions were as follows: initial denaturation in one cycle of 15 min at 95°C, followed by 40 cycles at 95°C for 20 s, 60°C (*β*-Actin) or 62°C (KLK14) for 20 s and 72°C for 30 s. Reaction, data acquisition and analysis were all performed by using the LightCycler® instrument. Gene expression was quantified by the comparative *C*_T_ method, normalizing *C*_T_-values to the housekeeping gene *β*-Actin and calculating the relative expression values of tumour and normal tissues (Fink *et al*, 1998).

### Anti-KLK14 antibody generation and cross-reactivity analysis

Purified recombinant human Kallikrein 14 (100 *μ*g), produced as the mature enzyme form in the *Pichia pastoris* expression system as previously described (Felber *et al*, 2005), was used as an immunogen and injected s.c. into New Zealand White female rabbits for polyclonal antibody development. The protein was diluted 1:1 in complete Freund's adjuvant for the first injection and in incomplete Freund's adjuvant for subsequent injections. Injections were repeated six times at 3-week intervals. Blood was drawn from the animals and tested for antibody generation every 2 weeks.

**Figure 1:**
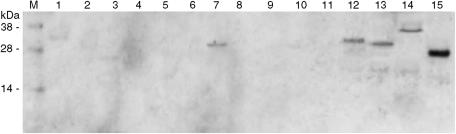
Specificity of the *α*-KLK14 rabbit polyclonal antibody. Purified natural or recombinant KLKs 1–13 and 15 (1 *μ*g; lanes 1 through 14, respectively) and recombinant KLK14 (100 ng; lane 15) were separated by SDS-PAGE, transferred to a nitrocellulose membrane and probed with *α*-KLK14 rabbit polyclonal antibody (1:2000). At this 1:10 weight ratio between KLK14 and other KLKs, this *α*-KLK14 rabbit polyclonal antibody can weakly detect KLKs 7, 12, 13 and 15 (lanes 7, 12, 13 and 14, respectively), as demonstrated by the relatively low intensity bands. This corresponded to <1% crossreactivity at a 1:1 weight ratio between KLK14 and other KLKs, as determined by semiquantitative densitometry. M, molecular weight marker.

Since KLK family proteins share 30–50% sequence similarity (Yousef and Diamandis, 2001), the potential crossreactivity of the *α*-KLK14 polyclonal rabbit antibody to other KLKs was evaluated by Western blotting followed by densitometry of resultant bands. Briefly, natural (KLK3) or recombinant (KLK1, 2, 4–15) hK proteins produced in-house (100 ng of KLK14; 1 *μ*g of other KLKs) were separated on 4–12% gradient polyacrylamide gels at 200 V for 30 min on the NuPAGE Bis-Tris electrophoresis system (Invitrogen, Carlsbad, CA, USA). Proteins were then transferred onto a Hybond-C extra nitrocellulose membrane (Amersham Biosciences, Piscataway, NJ, USA) at 30 V for 1 h. The membrane was blocked in Tris-buffered saline-Tween (0.1 mol l^−1^ Tris-HCl buffer (pH 7.5) containing 0.15 mol l^−1^ NaCl and 0.1% Tween 20) supplemented with 5% nonfat dry milk overnight at 4°C. The membrane was subsequently probed with the *α*-KLK14 polyclonal rabbit antibody (diluted 1:2000 in Tris-buffered saline-Tween with 5% nonfat dry milk) for 1 h at room temperature. After washing the membrane three times for 15 min and three times for 5 min with Tris-buffered saline-Tween, it was treated with an alkaline phosphatase (ALP) conjugated goat anti-rabbit IgG antibody (1:20 000 in Tris-buffered saline-Tween with 5% milk; Jackson Immunoresearch Inc., Pennsylvania, PA, USA) for 1 h at room temperature. The membrane was washed again as above and ALP activity was detected on X-ray film using a chemiluminescent substrate (Diagnostic Products Corporation, Los Angeles, CA, USA). Developed films were scanned and resultant band intensities were analysed by semiquantitative densitometry with Labworks™ 4.0 Image Acquisition and Analysis Software (Ultra-Violet Products Ltd., Upland, CA, USA). The *α*-KLK14 polyclonal rabbit antibody exhibited less than 1% crossreactivity at a 1:1 weight ratio between KLK14 and other KLKs (Figure 1).

### Immunohistochemistry

Formalin-fixed paraffin embedded tissue was freshly cut (4 *μ*m). The sections were mounted on superfrost slides (Menzel Gläser, Braunschweig, Germany), dewaxed with xylene and gradually hydrated. Antigen retrieval was achieved by pressure cooking in 0.01 M citrate buffer for 5 min. The KLK14 antibody was diluted 1:1000 using a background reducing dilution buffer (DAKO, Hamburg, Germany). No other blocking agents were employed. The primary antibody was incubated at room temperature for 1 h. As a negative control, four slides were processed without primary antibody. Detection was achieved by the conventional labelled-streptavidin-biotin (DAKO, Hamburg, Germany) method with ALP as the reporting enzyme according to the manufacturer's instructions. Fast-Red (Sigma-Aldrich, Munich, Germany) served as chromogen. Slides were briefly counterstained with haematoxylin and mounted.

### Evaluation of the immunohistochemical stainings

The immunostainings were independently examined by three pathologists, who were blinded to patient outcome. The scoring system for KLK14 staining was relatively simple so as to minimize interobserver variability and enhance reproducibility in future studies. Invasive carcinoma, intraductal carcinoma and adjacent normal breast tissue were evaluated on each slide. We evaluated the cytoplasmic staining intensity of KLK14 together with the percentage of cells stained. An IRS was applied, as described by Remmele and Stegner (1987). The IRS is the product of staining intensity (graded between negative=0 and strong=3) and the percentage of positively stained cells (graded between 0 and 4, being 1=<25%, 2=25–50%; 3=51–75%, and 4=>75%, respectively). Cases with discrepancies in IRS score among pathologists were discussed until consensus was reached.

### Statistical analysis

The data were analysed with the software package SPSS, version 12.0 (SPSS Inc., Chicago, USA). In order to compare the delta CT values of the real-time RT-PCR results between specific groups the nonparametric Mann–Whitney *U*-test was used. Fisher's exact and *χ*^2^ tests for trends were used to assess the statistical significance of associations between KLK14 expression and clinico-pathological parameters. Bivariate correlations according to Spearman were applied to the IRS of normal tissue, intraductal and invasive carcinomas. Univariate survival analysis was performed according to Kaplan–Meier and differences in survival curves were assessed with the Log rank test. *P*-values <0.05 were considered significant.

## RESULTS

### *Human kallikrein 14 (KLK14)* mRNA expression

**Figure 2:**
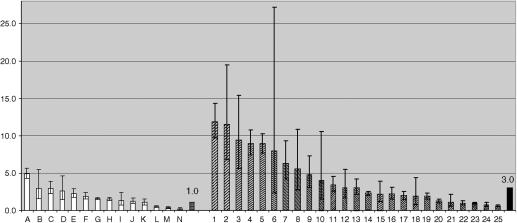
Diagrammatic presentation of quantitative RT-PCR data for *KLK14* mRNA from formalin-fixed paraffin-embedded breast cancer (samples 1–25) and normal breast tissue specimens (samples A–N). Mean *KLK14* expression was 3.0-fold upregulated in breast tumours as compared to the mean expression in normal breast tissues (set equal to 1).

*KLK14* mRNA expression was analysed by LightCycler® RT-PCR in a set of archival formalin-fixed paraffin-embedded tissue specimens, consisting of 25 primary breast cancer (14 from node-positive tumours, 11 from node-negative tumours) and 14 normal breast tissue samples. These data are presented in Figure 2. In 40% (10 out of 25) of the breast tumours analysed, we detected an at least two-fold upregulation in KLK14 mRNA expression compared to the mean KLK14 expression in normal breast tissue. Altogether, mean *KLK14* mRNA expression in breast tumours was 2.3-fold more abundant compared to the mean expression of KLK14 mRNA in normal breast tissues; this difference was statistically significant (*P*=0.027, two-tailed Mann–Whitney *U*-test). In this data set no significant association was found between *KLK14* expression level and histological grading, nodal status, tumour size or HER2, oestrogen and progesterone receptor status.

### Human kallikrein 14 immunostaining of breast tissue

**Figure 3:**
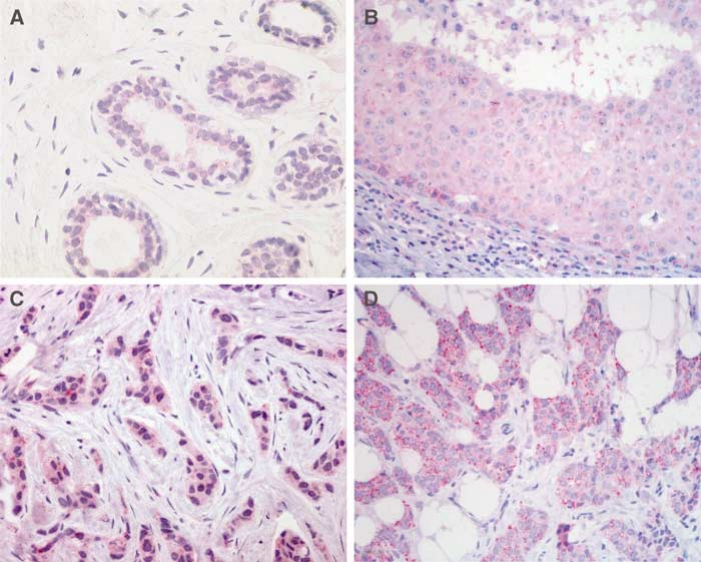
KLK14 immunohistochemistry in breast tissues. (**A**) Weak cytoplasmic immunoreactivity in secretory epithelia of normal lobular breast tissue. (**B**) Strong immunostaining of ductal carcinoma *in situ*. (**C** and **D**) Strong immunostaining in invasive ductal carcinomas with a diffuse cytoplasmic (**C**) and slightly granular pattern (**D**).

KLK14 was expressed in a weak to intermediate fashion in 91% of adjacent normal breast tissue (Figure 3A, Table 3). Intraductal carcinomas adjacent to the invasive tumour were present in 77 of the 127 cases. We observed cytoplasmic KLK14 expression in 99% of intraductal carcinomas (Figure 3B, Table 3) and in 96% of invasive carcinomas, respectively (Figure 3C and D, Table 3), which was significantly stronger than in normal tissue (*P*=0.003, Fisher's exact test). The IRS score in adjacent normal tissue (median IRS=2), intraductal (median IRS=4) and invasive carcinomas (median IRS=4) was significantly correlated (all *P*<0.001, Table 3). This implies that cases with higher IRS scores in normal adjacent tissues were likely to have higher IRS scores in the tumour. To correct for interindividual staining qualities, we normalized the KLK14 immunoreactivity in carcinomas by subtracting the IRS of the normal tissue from the corresponding tumour IRS. Cases in which the tumour stained stronger than adjacent normal tissue (IRS>=1) were considered as tumours with high KLK14 expression. KLK14 was shown to be overexpressed in 61% (78 out of 127 cases) of invasive breast carcinomas compared to the adjacent normal breast tissues. In 18% (23 out of 127 cases) and 21% (26 out of 127 cases) KLK14 expression was equal and higher in adjacent normal tissue compared to invasive carcinoma, respectively. There was a significant association of high KLK14 expression with higher tumour grade (*P*=0.041) and positive nodal status (*P*=0.045). KLK14 expression was not significantly associated with any other clinico-pathological parameters as shown in Table 4.

### Kallikrein 14 expression and survival

**Figure 4:**
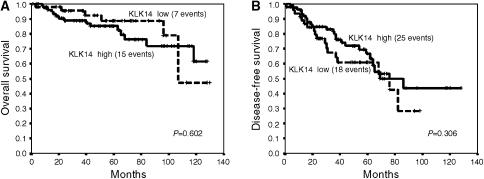
Univariate survival curves (Kaplan–Meier) for overall (**A**) and disease-free (**B**) survival according to high (bold line) versus low (dotted line) KLK14 expression. The number patients in the high and the low expression group were 78 and 49, respectively. The number of events in each group is stated in brackets.

Histological grade, tumour size and nodal status were significant predictors of both overall and disease-free survival (Tables 5, 6 and 7). However, KLK14 expression was not associated with either disease-free or overall patient survival. Kaplan–Meier survival curves (Figure 4A and B) indicate that patients with KLK14-negative tumours tended to exhibit a slightly increased overall survival compared to patients with KLK14-positive tumours; however, these results did not reach statistical significance. As previous results related KLK14 to steroid response, we performed survival analyses for KLK14 expression stratified for oestrogen receptor status and endocrine therapy but did not find any significant results for neither disease-free or overall survival (data not shown).

## DISCUSSION

In the present study, we carefully analysed KLK14 expression in normal and malignant breast tissue both on the RNA and protein level to further evaluate the potential value of KLK14 as a diagnostic marker in breast cancer. Using real-time RT-PCR we could clearly demonstrated KLK14 mRNA overexpression in breast cancer compared to normal breast tissue. These data are in conflict to a recent RT-PCR study, which showed loss of KLK14 expression in 21 of 25 (84%) human breast cancer analysed (Yousef *et al*, 2001). However, our data are in agreement with another quantitative RT-PCR study that described much more abundant KLK14 expression (in 55 of 178 breast tumours) and a clear correlation of KLK14 expression and poor prognosis (Yousef *et al*, 2002a). The possible explanation for these disparities could be that our study and that of Yousef *et al* (2002a) used the more accurate quantitative RT-PCR technique.

The present study is the first to analyse KLK14 protein expression in a large cohort of human breast cancer specimens in comparison to matching normal breast tissues using a recently characterised KLK14-specific antibody (Felber *et al*, 2005). This KLK14 antibody demonstrated KLK14 protein overexpression in 61% of analysed tumour/normal tissue pairs. Next, we analysed the KLK14 protein expression data in relation to established prognostic indicators and patient survival and we found a significant correlation of KLK14 protein expression with tumor grade and nodal status. Currently oestrogen receptor- and cerbB2-status represent the most important immunohistochemical diagnostic markers in breast cancer. Widely used, they provide clinically important information about the tumour biology and offer additional treatment options. This lead to a more individualized cancer therapy with antihormonal drugs or trastuzumab antibody (Fuqua and Cui, 2002; Bell *et al*, 2004), both of which improved patient outcome in selected patient cohorts. It remains to be focus of further study, whether KLK14 offers independent additional information in breast cancer or if KLK14 could be a potential target of specific therapy.

An earlier study by Yousef *et al* (2002a) examined the prognostic value of *KLK14* expression in 178 breast cancer on the RNA level and found that high *KLK14* mRNA expression was associated with advanced stage (III) disease. *KLK14* also was an independent predictor of decreased disease-free and overall survival. The cutoff value in this study (Yousef *et al*, 2002a) for KLK14 positive and negative tumours was defined by its ability to predict disease-free and overall survival and resulted in the definition of KLK14 positivity in 31% of breast tumours. In our study, we decided to use the median of KLK14 expression to delineate groups of low and high expression levels. With this cutoff value a significant association between KLK14 and tumour grading and positive nodal status was shown, both of which are indicators of a more aggressive course of disease. Alterations in the cutoff values to adapt the KLK14 postitive/negative ratio to the aforementioned study did not lead to any significant results (data not shown).

Borgono *et al* (2003) have previously proposed that KLK14 may represent a new biomarker for breast cancer, having shown KLK14 serum levels to be elevated in eight out of 20 (40%) breast cancer patients. This equals the KLK14 elevation in breast cancer tissues we observed in 40 and 61% of cases on RNA and protein level, respectively, and thus may account for the KLK14 elevation in the serum of a proportion of breast cancer patients.

Although the potential pathobiological role of KLK14 in breast cancer has not been elucidated, several lines of evidence suggest that KLK14, like many other kallikreins, may be causally involved in breast tumour progression (Borgono and Diamandis, 2004; Felber *et al*, 2005). This is substantiated by the fact that KLK14, via its serine protease activity, is able to degrade components of the extracellular matrix, including collagen IV *α*1 chain, collagen XII and matrilin-4 *in vitro*, which may promote tumour cell invasion and metastasis *in vivo* (Felber *et al*, 2005). These findings may have therapeutic applications by targeting KLK14 in appropriate patients.

In conclusion, although our data provides further evidence to support the association of KLK14 with aggressive breast cancer, immunohistochemically determined KLK14 expression in primary breast cancer does not appear to have additional prognostic value for the surgical pathologist. Further basic and clinical studies are warranted to clarify the potential role of KLK14 in the progression of breast cancer.

## Figures and Tables

**Table 1:** Clinicopathological parameters of the breast cancer patients

**Variable**	**Characteristic**	**Number of cases**	**%**
**Total number**		**127**	**100**
Histology	Invasive ductal	114	89.8
	Invasive lobular	13	10.2
			
pT status	pT1	84	66.1
	pT2	33	26.0
	pT3	4	3.1
	pT4	6	4.7
			
Nodal status	pN0	61	48.0
	pN1	30	23.6
	pN2	14	11.0
	pN3	19	15.0
	pNx	3	2.4
			
Grade	G1	29	22.8
	G2	64	50.4
	G3	34	26.8
			
Age	<60	72	56.7
	>=60	55	43.3
Adjuvant	None/radiotherapy	29	22.8
Therapy	Chemotherapy only	20	15.7
	Tamoxifen/±chemotherapy	70	55.1
			
Oestrogen	Negative	35	27.6
Receptor	Positive	80	63.0
c-erbB2	0, 1+	77	60.6
	2+, 3+	29	22.8

**Table 2:** Primers and probes used in real-time RT-PCR

**Gene**	**Primer sequence**	**Product size**
KLK14	5′-AGTGTCAGGCTGGGGAACTA-3′	183 bp
	5′-CCCAGAGTCACCCTGACAAG-3′	
		
*β*-Actin	5-GGACGACATGGAGAAAATC-3′	185 bp
	5-ATAGCACAGCCTGGATAGC-3′	

**Table 3:** KLK14 staining, immunoreactive score (IRS) and bivariate correlations in normal breast tissue, intraductal and invasive carcinoma

**Type of tissue**	**Percentage of tissue stained (n)**	**IRS median**	**IRS mean**	**Correlation with invasive carcinoma**
Normal breast (*n*=127)	91 (115)	2	2.9	CC=0.361
				*P*<0.001
Intraductal carcinoma (*n*=77)	99 (76)	4	5.1	CC=0.757
				*P*<0.001
Invasive carcinoma (*n*=127)	96 (125)	4	4.2	

CC=Correlation coefficient and *P*=*P* value.

**Table 4:** Associations between KLK14 expression and clinical-pathological parameters

		**No. of patients (%)**		
**Variable**	**Patients**	**KLK14 low**	**KLK14 high**	***P*-value**
*Patient age*				0.582
<60 years	72	46 (63.9)	26 (36.1)	
>=60 years	55	32 (58.2)	23 (41.8)	
				
*Histology*				0.246
Ductal	114	72 (63.2)	42 (36.8)	
Lobular	13	6 (46.2)	7 (53.8)	
				
*pT status*				0.323^a^
pT1	84	48 (57.1)	36 (42.9)	
pT2	33	24 (72.7)	9 (27.3)	
pT3/4	10	6 (60.0)	4 (40.0)	
				
*PN status*				0.045
pN0	61	29 (47.5)	32 (52.5)	
pN1+	64	19 (29.7)	45 (70.3)	
				
*Histological grade*				0.041
G1/2	93	41 (44.1)	52 (55.9)	
G3	34	8 (23.5)	26 (76.5)	
				
*Oestrogen receptor*				0.211
Negative	35	10 (28.6)	25 (71.4)	
Positive	80	34 (42.5)	46 (57.5)	
				
*CerbB2 expression*				1
0, 1+	77	31 (40.3)	46 (59.7)	
2+, 3+	29	11 (37.9)	18 (62.1)	
				
*Therapy*				0.563
None/local/CTx	49	20 (40.8)	29 (59.2)	
Tamoxifen±CTx	70	24 (34.3)	46 (65.7)	

a *χ*^2^ test for trends.

**Table 5:** Disease-free and overall survival according to clinicopathological variables and kallikrein 14 expression (univariate analysis)

	**Disease-free survival**				**Overall survival**			
**Characteristic**	**No. of cases**	**No. of events**	**5-year survival rate (±s.e.)**	***P*-value**	**No. of cases**	**No. of events**	**5-year nonprogression rate (±s.e.)**	***P*-value**
*KLK14 expression*				0.306				0.602
Low	49	18	64.5±6.9		49	7	89.0±4.4	
High	78	25	64.7±7.6		78	15	83.1±5.5	
								
*Age*				0.163				0.623
<60 years	72	28	55.9±7.1		72	14	88.6±4.1	
>=60 years	55	15	77.3±6.1		55	8	86.8±5.1	
								
*Histology*				0.929				0.856
Ductal	114	39	61.2±15.8		114	20	86.0±3.7	
Lobular	13	4	65.1±5.3		13	2	90.9±8.7	
								
*pT status*				0.001				0.001
pT1	84	21	76.9±5.4		84	6	94.6±2.6	
pT2	33	18	33.4±11.1		33	11	75.2±9.0	
PT3/4	10	4	60.0±15.5		10	5	60.0±19.3	
								
*Nodal status*				0.0295				0.001
PN0	61	13	78.5±6.3		61	2	96.2±2.6	
PN1+	64	28	55.1±7.3		64	20	77.7±5.7	
								
*Histological grade*				0.0155				0.043
G1/2	93	12	72.9±5.5		93	12	89.9±3.7	
G3	34	10	41.4±10.4		34	10	77.5±7.5	
								
*Oestrogen receptor*				0.7379				0.379
Negative	35	12	67.2±8.8		35	9	84.8±6.3	
Positive	80	27	64.5±6.6		80	12	85.2±4.6	
								
*c-erbB2 expression*				0.394				0.301
0, 1+	77	24	71.8±6.4		77	12	87.0±4.3	
2+, 3+	29	11	56.4±10.1		29	6	84.3±7.2	

**Table 6:** Cox univariate and multivariate analysis for disease-free survival. Variables were grouped according to Tables 4 and 5

**Variable**	**Relative risk**	**Confidence interval (95%)**	***P*-value**
*Univariate analysis*			
Kallikrein 14	1.370	0.746–2.517	0.310
pT status	1.441	1.044–1.990	0.026
Nodal status	2.479	1.450–4.237	0.001
Histological grade	2.539	1.543–4.178	<0.001
			
*Multivariate analysis*			
Kallikrein 14	0.572	0.292–1.119	0.103
pT status	0.845	0.495–1.442	0.536
Nodal status	2.078	0.942–4.584	0.070
Histological grade	2.159	1.033–4.514	0.041

**Table 7:** Cox univariate and multivariate analysis for overall-free survival. Variables were grouped according to Tables 4 and 5

**Variable**	**Relative risk**	**Confidence interval (95%)**	***P*-value**
*Univariate analysis*			
Kallikrein 14	1.269	0.516–3.120	0.604
pT status	2.150	1.394–3.316	0.001
Nodal status	4.313	1.654–11.250	0.003
Histological grade	2.521	1.265–5.022	0.009
			
*Multivariate analysis*			
Kallikrein 14	1.107	0.407–3.013	0.843
pT status	1.611	0.820–3164	0.166
Nodal status	4.434	0.889–22.106	0.069
Histological grade	1.391	0.512–3.777	0.517
